# The most primitive metazoan animals, the placozoans, show high sensitivity to increasing ocean temperatures and acidities

**DOI:** 10.1002/ece3.2678

**Published:** 2017-01-12

**Authors:** Dáša Schleicherová, Katharina Dulias, Hans‐Jűrgen Osigus, Omid Paknia, Heike Hadrys, Bernd Schierwater

**Affiliations:** ^1^ ITZ, Ecology and EvolutionTiHo HannoverHannoverGermany; ^2^ Present address: Department of Biological SciencesSchool of Applied SciencesUniversity of HuddersfieldHuddersfieldUK

**Keywords:** biomarkers, evolutionary constraints, global warming, ocean acidification, placozoa

## Abstract

The increase in atmospheric carbon dioxide (CO_2_) leads to rising temperatures and acidification in the oceans, which directly or indirectly affects all marine organisms, from bacteria to animals. We here ask whether the simplest—and possibly also the oldest—metazoan animals, the placozoans, are particularly sensitive to ocean warming and acidification. Placozoans are found in all warm and temperate oceans and are soft‐bodied, microscopic invertebrates lacking any calcified structures, organs, or symmetry. We here show that placozoans respond highly sensitive to temperature and acidity stress. The data reveal differential responses in different placozoan lineages and encourage efforts to develop placozoans as a potential biomarker system.

## 1 Introduction

Global warming has been changing the phenology, abundance, and distribution of many taxa in marine and terrestrial ecosystems (e.g., Falkowski, 2012; Thackeray, Jones, & Maberly, 2008) and ultimately affects all living taxa on earth. The immediate outcomes of climate change include ocean acidification, ocean warming, sea level rise (and subsequent changes in ocean circulation), and decrease in salinity (Houghton et al., 2001). For the potentially particularly affected benthic marine invertebrates, very little data exist and more empirical data are urgently needed in order to better understand possible changes in marine benthic ecosystems (Chen, 2008; Törnroos et al., 2014).

Animal populations may respond to shifting conditions in different ways, for example, expanding their ecological niche and/or by moving to a new habitat (Hinder et al., 2014). How such demographic processes will develop in the future has become a crucial question in many areas of ecological research. Habitat suitability models, which aim to predict how species ranges might change, are a theoretical means to find answers (e.g., Paknia & Schierwater, 2015; Törnroos et al., 2014). On the other side, empirical measures may include the use of sensitive biomarkers in long‐term monitoring studies and promise to be more sensitive and possibly also more reliable (cf. Feindt, Fincke, & Hadrys, 2014; Hadrys et al., 2005; Hardege et al., 2011; Schroth, Ender, & Schierwater, 2005).

### 1.1 Effects of ocean warming

Increasing temperatures often disturb physiological processes by damaging proteins, membrane fluidity, or organ function (Hochachka & Somero, 2002). As many marine organisms live close to their thermal tolerance (Hughes et al., 2003; Somero, 2002), increase in temperature may have severe impact on their performance and survival. Many reef‐building corals for example respond to warm episodes with widespread coral bleaching and show increased rates in mortality (Hughes et al., 2003; McWilliams et al., 2005). Often it is the planktonic larval or early benthic stages, which are especially sensitive (e.g., Foster, 1971; Pechenik, 1989). Rising water temperatures can also drive behavioral changes at the community level. To name just two out of many examples: The timing of spawning in the marine bivalve, *Macoma balthica*, is temperature dependent and so is the strength with which the sea star *Pisaster ochraceus* interacts with its principal prey (habitat forming mussels; Sanford, 1999). For the placozoans, which are found in most temperate and warm marine waters, nothing has been known yet about their sensitivity to temperature stress.

### 1.2 Effects of ocean acidification

The rapidly increasing carbonic emissions into the atmosphere (e.g., Neftel et al., 1985) have led to a decrease in the seawater pH at a rate of 0.02 units per decade (IPCC, 2013). This acidification can cause serious problems to organismal functions with respect to metabolism, calcification, and others (Langenbuch & Pörtner, 2003; Munday, Crawley, & Nilsson, 2009; Munday, Dixson, et al., 2009; Nakamura et al., 2011; Pörtner, 2008; Pörtner & Peck, 2010; Uthicke, Soars, Foo, & Byrne, 2013; Uthicke, Pecorino, et al., 2013). As a long‐term result, species communities may change, with some species simply disappearing (e.g., Goodwin et al., 2013; Pörtner, 2008) and others finding new niches (e.g., Foo et al., 2012; Parker et al., 2012; Sunday et al., 2011). No information is yet available for placozoans, which—in sharp contrast to the majority of other invertebrates—lack any kind of organs for homeostatic regulation.

Overall, the literature on documented effects of rising temperature and acidity on marine invertebrates is limited, but nonetheless covers a broad spectrum of levels of observation and sensitive taxa (Table 1). The shown summary table documents how fragmentary our current is. Evolutionary constraints are part of every organism, but the limitations for adaptation to environmental change are hard to foresee. Moreover, little is known about combined effects of ocean warming and acidification on the development of marine invertebrates. Combined effects of such stressors are not necessarily cumulative, because both additive and antagonistic (stress decreasing if combined) effects are known (Byrne & Przeslawski, 2013; Folt et al., 1999). Such effects have been studied in corals, mollusks, echinoderms, and crustaceans, across different ontogenetic stages. Additive negative effects on fertilization or growth rate, respectively, have for example been reported from the coral, *Acropora tenuis*, (Albright & Mason, 2013) and the oyster, *Crassostrea gigas* (Parker, Ross, & O'Connor, 2010). Antagonistic effects have been found for example in the sea urchins *Heliocidaris tuberculata* (Byrne et al., 2010) and *Sterechinus neumayeri* (Byrne et al., 2013; Ericson et al., 2011), where warming partially compensated for the negative effect of acidification on larval growth.

**Table 1:** Summary of temperature and ocean acidification effects on marine biota in current literature

Major group	Studied organism	Effects of temperature	Effects of pH	Reference
Macroalgae	*Amphiroa fragillisima*		Decrease in calcification	Langdon et al. (2003)
	*Chondria dasyphylla*			
	*Gelidiopsis intricate*			
	*Haptilon cubense*			
	*Sargassum muticum* and *Cystoseira tamariscifolia*	Reduce in biomass of macroalgal assemblages	Reduce in biomass of macroalgal assemblages	Olabarria et al. (2013)
Cnidaria	*Acropora digitifera*		Reduced metabolic rates	Nakamura et al. (2011)
	*Stylophora pistillata*	Net photosynthesis affected	Cell‐specific density affected	Reynaud et al. (2003)
	*Aiptasia pulchella*	Host cell adhesion dysfunction		Gates, Baghdasarian, and Muscatine (1992)
	*Pocillopora damicornis*			
	*Diploria strigosa*	Negative effect on larval development		Bassim, Sammarco, and Snell (2002)
Bryozoa	*Membranipora membranacea*	Capable of acclimating to elevated temperatures		Menon (1972)
	*Electra pilosa*			
	*Conopeum reticulum*			
	*Myriapora truncata*	Negative effect on calcification (combination of temperature rise and ocean acidification)	Negative effect on calcification (combination of temperature rise and ocean acidification)	Rodolfo‐Metalpa et al. (2010)
			Corrosion of calcareous skeletons	Lombardi et al. (2011)
Mollusks	*Clio pyramidata*		Reduced calcification rates	Fabry et al. (2008)
	*Crassostrea gigas*		Calcification rates decrease	Gazeau et al. (2007)
	*Haliotis laevigata*		Affected specific growth rate	Harris et al. (1999)
	*Haliotis rubra*			
	*Mercenaria mercenaria*		Dissolution‐induced mortality	Green et al. (2004)
	*Mytilus edulis*		Negative effects on growth	Berge et al. (2006)
			Calcification rates decrease	Gazeau et al. (2007)
	*Saccostrea glomerata*		Possibility to adapt	Parker et al. (2012)
		Decreased fertilization	Abnormal D‐veligers	Parker, Ross, and O'Connor (2009)
	*Strombus luhuanus*		Affects growth	Shirayama and Thornton (2005)
Arthropods	*Acartia clausi*	Respiration and ammonia excretion		Gaudy, Cervetto, and Pagano (2000)
	*Acartia erythraea*		Reproduction rate and larval development	Kurihara, Shimode, and Shirayama (2004)
	*Acartia steueri*			
	*Acartia tonsa*	Respiration and ammonia excretion		Gaudy et al. (2000)
	*Callinectes sapidus*		Compensation of hypercapnia	Cameron and Iwama (1987)
Echinoderms	*Acanthaster planci*		Negative impacts on larval development	Uthicke, Soars, et al. (2013), Uthicke, Pecorino, et al. (2013)
	*Centrostephanus rodgersii*	Decrease in gastrulation	Decrease in cleavage stage embryos	Foo et al. (2012)
	*Echinometra mathaei*		Early development	Kurihara and Shirayama (2004)
			Affects growth	Shirayama and Thornton (2005)
			Male spawning ability	Uthicke et al. (2013)
	*Hemicentrotus pulcherrimus*		Early development	Kurihara and Shirayama (2004)
			Affects growth	Shirayama and Thornton (2005)
	*Pisaster ochraceus*	Affects keystone predation		Sanford (1999)
	*Psammechinus miliaris*		Hypercapnia and mortality	Miles et al. (2007)
	*Strongylocentrotus franciscanus*		Thermal stress	O'Donnell et al. (2009)
Chordata	*Amphiprion percula*		Impairs olfactory discrimination	Munday, Crawley, et al. (2009), Munday, Dixson, et al. (2009)
	*Ictalurus punctatus*		Compensation of hypercapnia	Cameron and Iwama (1987)
	*Lepidonotothen kempi*		Inhibition of protein biosynthesis	Langenbuch and Pörtner (2003)
	*Ostorhinchus cyanosoma*	Declines in aerobic scope	Declines in aerobic scope	Munday, Crawley, et al. (2009), Munday, Dixson, et al. (2009)
	*Ostorhinchus doederleini*			
	*Pachycara brachycephalum*		Inhibition of protein biosynthesis	Langenbuch and Pörtner (2003)
	*Sillago japonica*		Acute toxicity on juveniles	Kikkawa et al. (2006)

In this study, we investigate the effects of temperature and acidity stress on placozoan reproduction and report strong and differential effects for both factors on the population growth rate (PGR) in different lineages (species) of placozoans. The observed differential sensitivity of different placozoan species or lineages suggests that placozoans might be promising organisms for developing a new generation of biomonitoring systems.

## 2 Materials and Methods

### 2.1 Study organism

The phylum Placozoa holds a key position in the metazoan Tree of Life, close to the last common metazoan ancestor. Placozoans represent the simplest (not secondarily reduced) metazoan bauplan and have become an emerging model organism for understanding early metazoan evolution (Eitel et al., 2013; Schierwater, de Jong, & DeSalle, 2009; Schierwater, Eitel, et al., 2009; Schierwater et al., 2016; Signorovitch, Dellaporta, & Buss, 2006).

These tiny invertebrates are common in warm tropical and subtropical as well as in some temperate marine waters in different depths up to 20 m. Their preferred habitats are calm water areas with hard substrates like mangrove tree roots, rocks, corals, and other hard substrates in the eulittoral and littoral zone. Placozoans have occasionally also been found on sandy surfaces or in areas with high wave activity. Yet, the biodiversity and ecology of placozoans are poorly known (Eitel & Schierwater, 2010; Maruyama, 2004; Pearse & Voigt, 2007).

Recent genetic studies have revealed a high biodiversity and systematic complexity of the Placozoa. As no morphological differences are visible among placozoan haplotypes in light microscopy, the known haplotypes represent “cryptic” species (Eitel & Schierwater, 2010; Loenarz et al., 2011; Schierwater, 2005; Schierwater, de Jong, et al., 2009; Schierwater, Eitel, et al., 2009; Signorovitch et al., 2006). At present, the phylum Placozoa is the only monotypic phylum in the animal kingdom, with the only formally described species *Trichoplax adhaerens* (Schulze, 1883, 1891). Placozoans offer unique possibilities for experimental ecophysiological studies because of their small size, simple morphology, and fast vegetative reproduction (Eitel & Schierwater, 2010; Eitel et al., 2011, 2013; Schierwater, 2005). Vegetative reproduction through binary fission or budding is the usual mode of reproduction in the laboratory and in the field. In contrast, bisexual reproduction is rarely seen in the laboratory, but most likely present in all placozoans (Eitel et al., 2011; Signorovitch, Buss, & Dellaporta, 2007). The details of sexual reproduction and embryonic development in placozoans remain widely unknown, because all efforts to complete the sexual life cycle in the laboratory have been unsuccessful, because embryonic development has never gone beyond the 128 cell stage (Eitel et al., 2011). As the overall effects of physiological stress are best seen in the performance of vegetative reproduction by binary fission, we used overall PGR as the dependent and easily quantifiable variable for the subsequent experiments.

### 2.2 Experimental setup for temperature experiments

All animal lineages used in the experiments have been cultured in our Institute of Animal Ecology and Cell Biology of the TiHo, Hannover (Germany), for several years:

1. H1*—Trichoplax adhaerens* (cosmopolitic), our so‐called Grell lineage found by Karl Gottlieb Grell in an algal sample from the Red Sea in 1969, hereafter named “H1_gre_.” For 30 years, this lineage had been cultured in Bochum (Wenderoth & Ruthmann laboratory), and in 1999, it was transferred to the Schierwater laboratory (Schierwater, 2005).
2. H2—“Roscoff” (cold‐water population): This haplotype derived from a single animal collected from the coast of Roscoff (France) in 2009 and is hereafter named “H2_ros_” (von der Chevallerie, Eitel, & Schierwater, 2010).
3. H2—“Panama” (warm‐water population): This haplotype culture derived from a single animal collected in 2002 in Bocas del Toro (Panama), hereafter named “H2_pan_” (Eitel et al., 2013).

H1 and H2 represent different species (Schierwater, Osigus, Kamm K, Eitel M, & DeSalle, in preparation), while the two H2 lineages are different populations of the same species.

**Figure 1:**
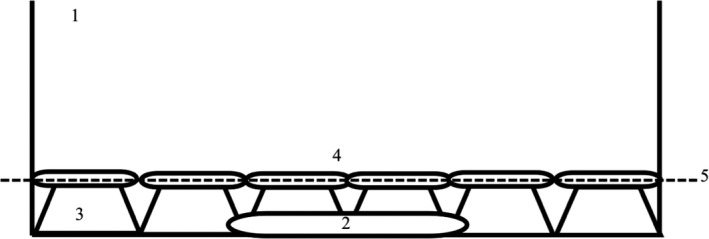
The experimental setup for the temperature experiment. 1—Aquarium filled with artificial seawater, 2—heater, 3—glass bowls turned over, 4—covered Petri dishes with the experimental animals placed on the glass bowls, 5—surface line of artificial seawater

All experiments were carried out in glass Petri dishes (Ø: 14 cm) placed at three different temperatures (low = 21°C, medium = 25°C, and high = 29°C). About 21°C (room temperature) was maintained in the laboratory by means of an air‐conditioning system (DC Inverter, Fujitsu). Experimental groups tested at 25 and 29°C were placed in separate aquaria (in the same room), filled with ASW (artificial seawater), and heated to the desired temperature by two heaters (ProTemp S200, accuracy: ±0.5°C). To keep the water temperature evenly distributed within aquaria, a water pump was installed to circulate the water (Figure 1).

At the start of the experiment, 360 individuals per lineage were randomly assigned to nine experimental groups (Table S1). Testing three lineages of placozoans, each for three different temperatures, we performed eight replicates with each five specimens as a starting point. After an acclimation period of 2 days (the chosen placozoan species adapt very quickly to new culture conditions), and in order to measure the PGR over the 3 weeks experimental period, the total number of individuals per plate was counted every 3 days (nine censuses).

### 2.3 Experimental setup for pH experiments

**Figure 2:**
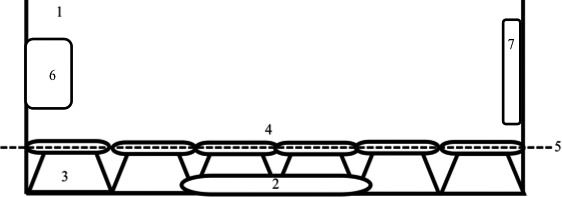
The experimental setup for the pH experiment. 1—Aquarium filled with artificial seawater, 2—heater, 3—glass bowls turned over, 4—covered Petri dishes with the experimental animals placed on the glass bowls, 5—surface line of artificial seawater, 6—CO_2_‐reactor, and 7—pH meter

We used the same lineages as described above. The aquarium was setup with a CO_2_ reactor (JBL ProFlora), a pH meter, and an aeration system for the seawater carbon dioxide (CO_2_) and the manipulation of the pH (for further details, see also Riebesell et al., 2000 and Figure 2).

At the start of the experiment, 80 specimens per lineage were randomly assigned to six experimental groups (Table S2). Food was provided ad libitum by placing one slide covered with algae inside the Petri dish. After an acclimation period of 2 days, the placozoans were left in one of two 160‐L aquaria, one with a constant pH of 7.6, and the other with a pH of 8.0 (control; normal pH conditions in the laboratory cultures). In order to measure the PGR during the experimental period (12 days), the total number of individuals per plate was counted every 2 days (five censuses).

### 2.4 Statistical analysis

The Kolmogorov–Smirnov one‐sample test was used to test for normality distribution. As none of the data sets showed normal distribution (Kolmogorov–Smirnov test; *p* < .05), the data were normalized by log‐transformation for the temperature experiment. Differences in PGR between the three different temperature settings were tested for by one‐way ANOVA with the total number of individuals as a dependent variable and treatment as a fixed factor. Differences in PGR between the two different pH settings were tested for by means of the Mann–Whitney *U*‐test. Thus, PGRs were compared between treatments (three different temperatures—experiment 1; two different pH—experiment 2) in the three clonal lineages (H1_gre_, H2_ros_, and H2_pan_). Statistical analyses of both experiments were performed using the statistical software Minitab 16 and PAST (Hammer, Harper, & Ryan, 2001). Descriptive statistics are reported as means ± *SE*

## 3 Results

Both factors, temperature and pH, affected the PGR of different placozoan lineages significantly.

### 3.1 The effect of temperature

**Figure 3:**
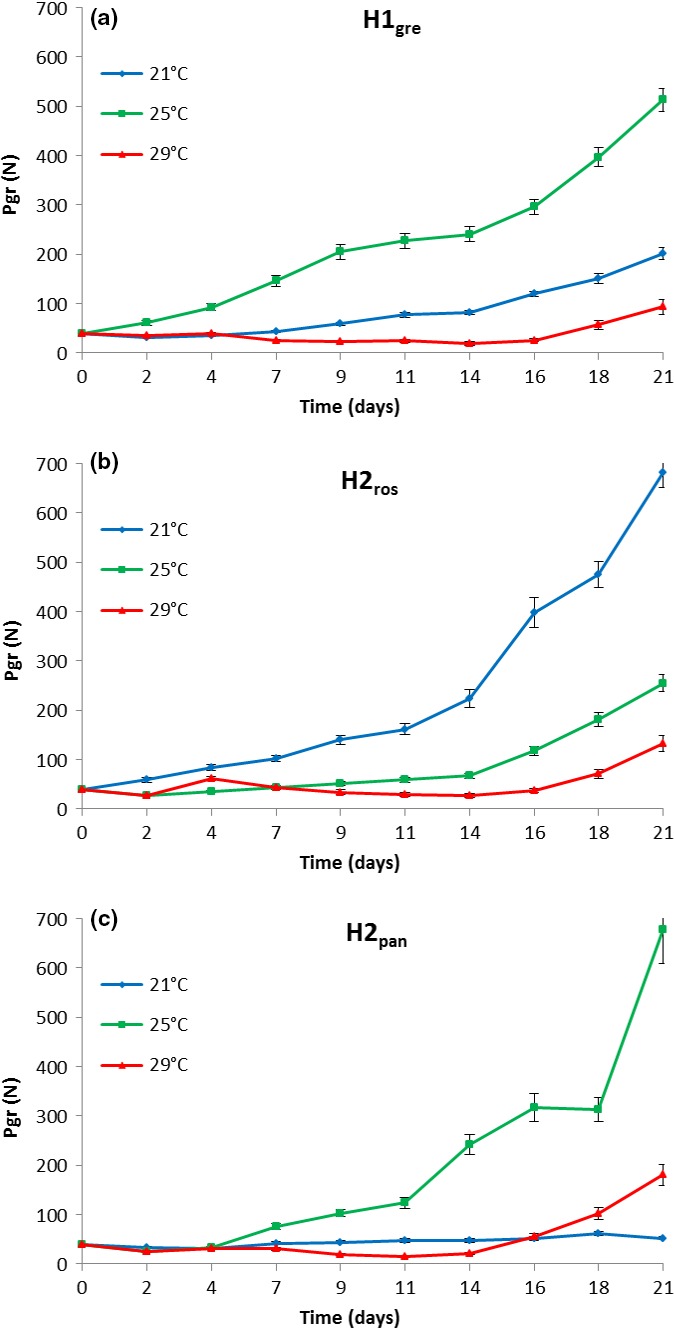
Population growth rate (PGR) at different temperatures for the three placozoan lineages (a) H1_gre_, (b) H2_ros_, and (c) H2_pan_

The three lineages H1_gre,_ H2_ros_, and H2_pan_ responded in sharply different ways to changes in water temperature:

1. The cosmopolitic H1_gre_:

One‐way ANOVA revealed highly significant differences in the PGR for the three different temperatures (*F*_2, 27_ = 14.89, *df* = 2, *p* < .001). Post hoc tests revealed highly significant differences in the PGR between 25 and 29°C (*p* < .001) and also between 21 and 25°C (*p* = .013). Between 21 and 29°C, no significant difference was observed (*p* > .05); at both temperatures, the PGR was low compared to the “optimal” temperature of 25°C (Figure 3a).

The cold‐water H2_ros_:

Also here, the effect of temperature on the PGR was significant (*F*_2, 27_ = 8.04, *df* = 2, *p* = .002; one‐way ANOVA). Post hoc tests revealed significant differences in the PGR between 21 and 29°C (*p* = .002) and also between 21 and 25°C (*p* = .033), while between 25 and 29°C, no significant difference was observed (*p* > .05). At both higher temperatures, the PGR of the cold H2_ros_ was low suggesting the lower temperature of 21°C to be preferred (Figure 3b).

The warm‐water H2_pan_:

The H2_pan_ clone behaved similar to the H1_gre_ clone, showing significant changes in PGR when moving away from the “optimal” temperature of 25°C (*F*_2, 27_ = 6.08, *df* = 2, *p* = .007; one‐way ANOVA). The harmful effect of higher temperature even on the warm‐water population seems particularly notable (Figure 3c).

**Figure 4:**
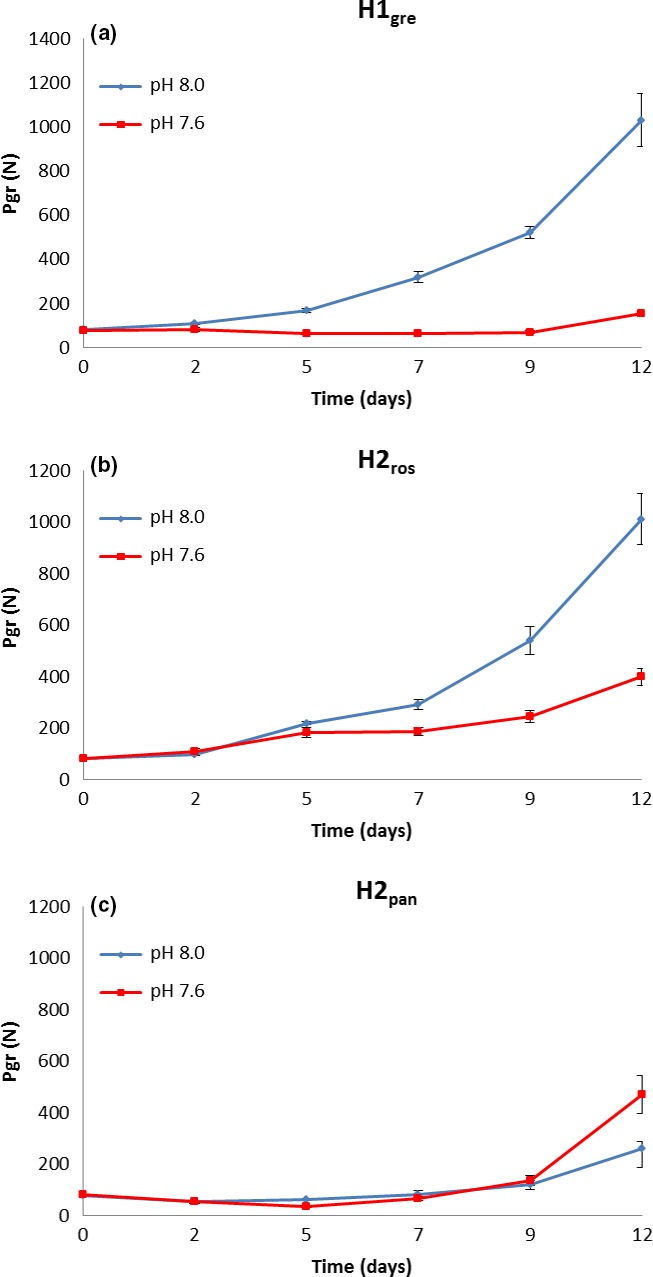
Population growth rate (PGR) at the two different pH levels for the lineages (a) H1_gre_, (b) H2_ros_, and (c) H2_pan_

Profound effects of slight changes in pH value were found for the lineages H1_gre_ and H2_ros_. After about 5 days into the experiment, the PGR in the acidified water slowed down significantly compared to the control (pH 8.0) cultures, with the effect becoming more and more substantial over time (Figure 4a–c and Table 2). The Panama lineage showed an unusual slow PGR under the given conditions (room temperature—21°C) already at “normal” pH conditions. As we do not know the reasons for the unusual slow reproductive activity, we excluded these data from further analyses. The observation that under more acid conditions, the PGR was higher than under pH 8.0 conditions maybe an artifact or may indeed be a lineage‐specific adaptive response, but at this point, any further conclusions would be premature.

**Table 2:** Influence of increased water acidity on the PGR in the placozoan lineages H1_gre_, H2_ros_, and H2_pan_ (Mann–Whitney *U*‐test at the different observation points; bold = significant values)

Lineage	Time (days)	*p* Value (>.050)	Monte Carlo *p*	Exact *p*
H1_gre_	2	.3123	.3461	.3429
H1_gre_	5	**.0294**	**.0289**	**.02857**
H1_gre_	7	**.0294**	**.0288**	**.02857**
H1_gre_	9	**.03038**	**.0323**	**.02857**
H1_gre_	12	**.03038**	**.0282**	**.02857**
H2_pan_	2	.8852	1	1
H2_pan_	5	**.0294**	**.0296**	**.02857**
H2_pan_	7	.8852	1	1
H2_pan_	9	.8852	.8877	.8857
H2_pan_	12	.3123	.3441	.3429
H2_ros_	2	.5614	.5405	.5429
H2_ros_	5	.3123	.3496	.3429
H2_ros_	7	.1124	.1153	.1143
H2_ros_	9		.0588	.05714
H2_ros_	12	.0606	.0546	.05714

PGR, population growth rate.

## 4 Discussion

Climate change is directly or indirectly affecting the distribution, abundance, breeding, and migration of marine plants and animals (e.g., Doney et al., 2009; Hoegh‐Guldberg & Bruno, 2010; Ji et al., 2007; Jiao et al., 2015). Mean global temperatures will continue to rise even if greenhouse gas emissions are stabilized at present levels (IPCC, 2001, 2013). Some of the most affected ecosystems are the oceans, which show rising temperature and acidity. Sensitive organisms, which respond to such changes early and are restrained from quick adaptations by evolutionary constraints, might be useful biomarkers for biomonitoring studies (e.g., Dallas & Jha, 2015; Moschino, Del Negro, & De Vittor, 2016; Natalotto et al., 2015) .

Our experiments revealed strong and differential effects of both, temperature and pH, on the PGR of placozoans, with temperature showing the strongest effects. Interestingly, but not surprisingly, the lineage which had been found in relatively cold Atlantic waters (H2_ros_) showed a thermal preference for the low temperature setting, whereas higher temperatures significantly reduced the PGR. The other two lineages performed best at 25°C, which has been regarded as the “normal” temperature for placozoans (Schierwater, 2005). Both, *T. adhaerens* (species H1_gre_, which has been collected from the Red Sea) and H2_pan_ (collected from Panama), only performed well at 25°C. Interestingly, for clones adapted to tropical waters, both species almost cease propagation at the high temperature of 29°C. As all clones sharply reduce propagation rates at the highest temperature, we assume harmful effects of such high temperatures for placozoans in general.

Placozoans behave like most marine species, which show thermal preferences for a well‐defined temperature range (IPCC, 2007; Nakano, 2014). In many locations, ocean temperatures have either increased (Bethoux, Gentili, & Tailliez, 1998; Freeland, 1990; IPCC, 1996, 2001, Ji et al., 2007; Scranton et al., 1987) or decreased in short time (IPCC, 1996, 2001, Ji et al., 2007; Read & Gould, 1992), and demographic effects on many marine species, including placozoans, must have occurred recently. According to Hiscock et al. (2004), the ocean temperature will continue to show significant short‐term variations, with maximum ocean‐surface temperatures close to 28°C (with a trend toward even higher temperatures). As the natural habitat of placozoans is mainly surface waters, we must predict ongoing demographic changes and differential effects on placozoan communities. Such differential effects mark placozoans as potential biomarkers for monitoring studies on the effects of ocean warming.

**Figure 5:**
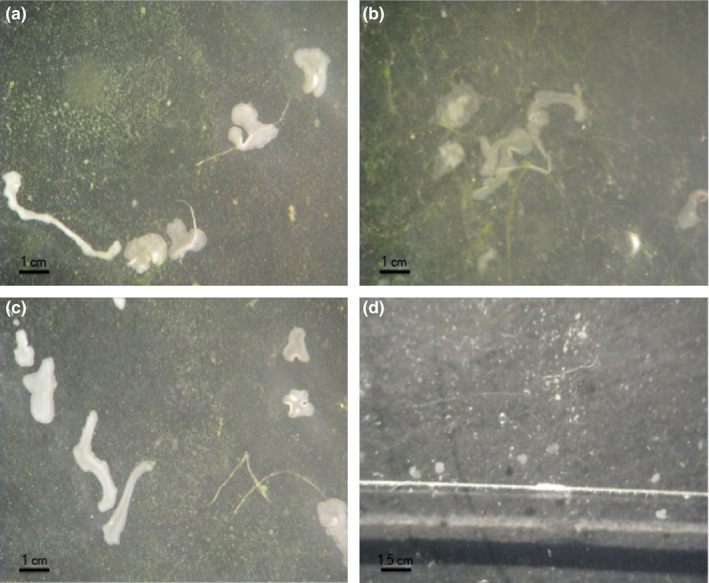
Changes in morphology of *Trichoplax adhaerens* under acidity stress. (a) Unusually enlarged specimens, (b) extremely long specimen, (c) normal to very long shaped specimens, (d) very tiny, round‐shaped specimens

The sharp decline in propagation rate observed in *T. adhaerens* (H1_gre_) and H2_ros_ mirrors a quite sensitive response to increasing water acidity. This sensitivity is also highlighted by quite extreme changes in morphology toward the end of the experiments (Figure 5). These dramatic and harmful effects forced us to end the experiments after 12 days. Although the experiments on the H2_pan_ clone were not conclusive, the relative increase in PGR toward the end of the experiment as well as the differences between the other two clones suggests that different placozoan lineages differ in their sensitivity and response to change in water acidity. These observations not only highlight the sensitivity of placozoans to water acidity but also point to the potential of combining different sympatric placozoan species into a multiple‐species biomarker system. Several other examples of sympatric species complexes might be available also from different other invertebrate taxa (e.g., Azevedo et al., 2015; Hoegh‐Guldberg & Bruno, 2010; Kroeker et al., 2013; Nakamura et al., 2011; Navarro et al., 2013; O'Donnell, Hammond, & Hofmann, 2009; Schmidt, Power, & Quinn, 2013).

As Malakoff (2012) points out, understanding the ecological and evolutionary implications of acidifying oceans requires extended experiments and long‐term monitoring studies. Kelly and Hofmann (2012) review empirical studies on adaptability and acclimatization of marine organisms to elevated *p*CO_2_ values (e.g., in algae, positive trends for photosynthesis were found), including adaptation reports from some cnidarians, which increase their biomass with increasing *p*CO_2_. What may look like a “favorable” response and quick adaptation here certainly does not apply to placozoans. Here, each factor by itself, temperature and acidity, can bring growth rate to die down and a combination of both factors must be even more detrimental. On the other hand, this sensitivity can open new avenues for using placozoans as sensitive biosensors in long‐term biomonitoring studies.

### 4.1 Final conclusions

Placozoans, the most simple organized and possibly also the oldest metazoan animals (cf. Schierwater, de Jong, et al., 2009; Schierwater, Eitel, et al., 2009), are highly sensitive to temperature and acidity stress and thus might be explored as potential biosensors. They offer the unique advantage of showing differential response patterns in different but sympatrically occurring placozoan species. The potential of a multiple “cryptic” species monitoring system has not been explored yet, but in practice should be based upon high‐throughput genetic assays of community diversity and stress gene expression. Furthermore, the quantified differences in niche parameters must also be relevant for species descriptions following the taxonomic circle approach in a large group of cryptic placozoan species.

## Conflict of Interest

None declared.
