# Supplementary material for: The most primitive metazoan animals, the placozoans, show high sensitivity to increasing ocean temperatures and acidities
Source: Ecol Evol. 2017 Jan 12;7(3):895–904. doi: 10.1002/ece3.2678 (PMC5288258; doi:10.1002/ece3.2678)
Supplement: Supplementary file 1 [file ECE3-7-895-s001.docx]

**Supplementary material**

Table S1. Experimental design of the Experiment 1: Test of the temperature effect on the PGR.

Table S2. Experimental design of the Experiment 2: Test of the acidification effect on the PGR.

Table S1.

| Lineage | T (°C) | Individuals set up/ Petri dish | Replicates (N) |
| --- | --- | --- | --- |
| H1_gre_ | 21 | 5 | 8 |
| H1_gre_ | 25 | 5 | 8 |
| H1_gre_ | 29 | 5 | 8 |
| H2_pan_ | 21 | 5 | 8 |
| H2_pan_ | 25 | 5 | 8 |
| H2_pan_ | 29 | 5 | 8 |
| H2_ros_ | 21 | 5 | 8 |
| H2_ros_ | 25 | 5 | 8 |
| H2_ros_ | 29 | 5 | 8 |

Table S2.

| Lineage | *p*CO_2_ (pH) | Individuals set up/ Petri dish | Replicates (N) |
| --- | --- | --- | --- |
| H1_gre_ | 8.0 | 20 | 4 |
| H1_gre_ | 7.6 | 20 | 4 |
| H2_ros_ | 8.0 | 20 | 4 |
| H2_ros_ | 7.6 | 20 | 4 |
| H2_pan_ | 8.0 | 20 | 4 |
| H2_pan_ | 7.6 | 20 | 4 |
